# Ultrasonic Sensor: A Fast and Non-Destructive System to Measure the Viscosity and Density of Molecular Fluids

**DOI:** 10.3390/bios14070346

**Published:** 2024-07-16

**Authors:** Romina Muñoz, Juan-Francisco Fuentealba, Sebastián Michea, Paula A. Santana, Juan Ignacio Martinez, Nathalie Casanova-Morales, Vicente Salinas-Barrera

**Affiliations:** 1 Departamento de Física y Química, Facultad de Ingeniería, Universidad Autónoma de Chile, Av. Pedro de Valdivia 425, Providencia, Santiago 8900000, Chile; romina.munoz@uautonoma.cl; 2 Escuela de Ingeniería, Universidad Central de Chile, Avda. Santa Isabel 1186, Santiago 8330601, Chile; juan.fuentealba@ucentral.cl; 3 Grupo de Investigación Aplicada en Robótica e Industria 4.0, Instituto de Ciencias Aplicadas, Facultad de Ingeniería, Universidad Autónoma de Chile, Santiago 7500912, Chile; sebastian.michea@uautonoma.cl; 4 Instituto de Ciencias Aplicadas, Facultad de Ingeniería, Universidad Autónoma de Chile, El Llano Subercaseaux 2801, San Miguel, Santiago 8910060, Chile; paula.santana@uautonoma.cl; 5 Ingeniería Civil Informática, Facultad de Ingeniería, Universidad Autónoma de Chile, Av. Pedro de Valdivia 425, Providencia, Santiago 8900000, Chile; juan.martinez2@cloud.uautonoma.cl; 6 Facultad de Artes Liberales, Universidad Adolfo Ibáñez, Santiago 7941169, Chile

**Keywords:** ultrasonics sensor, resonance system, biofluid applications, chemical compounds

## Abstract

This study presents the design and development of an ultrasonic sensor as a fundamental tool for characterizing the properties of fluids and biofluids. The analysis primarily focuses on measuring the electrical parameters of the system, which correlate with the density and viscosity of the solutions, in sample volumes of microliters and with high temporal resolution (up to 1 data point per second). The use of this sensor allows the fast and non-destructive evaluation of the viscosity and density of fluids deposited on its free surface. The measurements are based on obtaining the impedance versus frequency curve and the phase difference curve (between current and voltage) versus frequency. In this way, characteristic parameters of the transducer, such as the resonance frequency, phase, minimum impedance, and the quality factor of the resonant system, can characterize variations in density and viscosity in the fluid under study. The results obtained revealed the sensor’s ability to identify two parameters sensitive to viscosity and two parameters sensitive to density. As a proof of concept, the unfolding of the bovine albumin protein was studied, resulting in a curve that reflects its unfolding kinetics in the presence of urea.

## 1. Introduction

The necessity for monitoring fluids in real time within various industries has catalyzed the development of technologies that are both cost-effective and accurate. Challenges such as elevated costs and extensive maintenance requirements have prompted the scientific and industrial sectors to explore innovative, non-destructive methodologies for measuring fluid density and viscosity. These advancements respond to a critical demand for monitoring solutions that are both reliable and readily accessible.

Among the various techniques available, ultrasonic methods have been identified as particularly significant. These techniques utilize high-frequency sound waves for the detection of defects, dimension measurements, and the characterization of material properties with exceptional precision [1]. Capable of being implemented through either distinct transmitter and receiver units or a combined transceiver unit (pulse-echo mode), ultrasonic methods offer extensive applicability across numerous sectors. Specifically, in the oil and gas industry, ultrasonic testing is indispensable for pipeline inspection [2] and maintenance, enabling the detection of corrosion, cracks, and other discontinuities that could compromise pipeline integrity [3,4]. In the realm of medicine, ultrasonography serves as a non-invasive diagnostic tool, providing real-time visualization of internal organs, tissues, and blood flow [5,6], with particular significance in obstetrics for monitoring fetal development [7]. Moreover, in materials science and engineering, ultrasonic techniques are crucial for determining the mechanical properties of materials, detecting structural flaws, and evaluating weld quality [8].

The search for non-destructive techniques capable of measuring fluid viscosity and density has led to the development of various experimental setups and conditions. Notably, a device proposed by Abdulkareem et al. [9] features a cost-effective, disposable probe equipped with a piezoelectric sensor/actuator pair, designed for the real-time assessment of fluid viscosity. This device leverages wave propagation patterns to efficiently detect viscosity variations in small liquid volumes, such as those in vacutainer vials (>2 mL). Concurrently, Kasys et al. [10] introduced an advanced ultrasonic technique for the precise measurement of liquid densities under extreme in situ conditions. They demonstrated its applicability across a diverse range of materials, including liquids and polymer melts, and its resilience to temperature fluctuations.

Concurrently, Kasys et al. [10] introduced an advanced ultrasonic technique for the precise measurement of liquid densities under extreme in situ conditions, demonstrating its applicability across a diverse range of materials, including liquids and polymer melts, and its resilience to temperature fluctuations.

The measurement of viscosity holds significant importance within biological systems, finding applications in biochemistry, biomedicine, pharmacy, and the food and cosmetics industries [11]. Variations in viscosity can profoundly impact intracellular transport, biomolecule interactions, and the diffusion of metabolites in living organisms. Furthermore, abnormal viscosity levels in biological samples, such as blood, urine, and saliva, often serve as indicators of various diseases, including cardiovascular disease, diabetes, and Alzheimer’s disease [12,13]. The stability of protein-based therapeutics, which is crucial for drug efficacy and patient safety, can also be evaluated through viscosity measurements [14,15].

However, the measurement of viscosity in biological solutions poses challenges, primarily due to the large sample volumes required by conventional rheometers and the shear applied during testing, which can alter the integrity of macromolecules under study [16]. Recent advancements have sought to mitigate these issues by reducing the required sample volume and minimizing shear effects, thus preserving the structural and functional integrity of sensitive biomolecules [17,18,19]. Emerging techniques, such as passive rheology and resonant acoustic rheometry, offer innovative approaches for assessing the mechanical properties of soft and viscoelastic materials with minimal sample disturbance [20,21].

This paper highlights the use of a single ultrasonic transducer emitting low-amplitude ultrasonic waves for evaluating the properties of fluid droplets placed on the radiating face of the transducer. This produces changes in the characteristic parameters of the electrical response of the transducer, such as resonance frequency, phase change, quality factor, and minimum impedance of the transducer-droplet system, which are related to fluid properties such as density and viscosity. The system is capable of detecting changes even with sample volumes of less than 1 mL. Our initial investigations have established an evaluation between specific observable variables and the properties of glycerol and polyethylene glycol solutions at known concentrations. Furthermore, the application of the technique in biological samples has been considered a proof of concept by measuring the evolution of a solution of the bovine serum albumin protein with urea, which promotes its unfolding [22] and, as a consequence, changes in its viscosity [23]. The system was optimized to acquire values of electrical parameters with a frequency of up to 1 data point every 10 s, demonstrating its potential to detect small viscosity variations with high temporal resolution, thus opening new opportunities for research and diagnostic applications.

## 2. Materials and Methods

### 2.1. Experimental Setup

**Figure 1:**
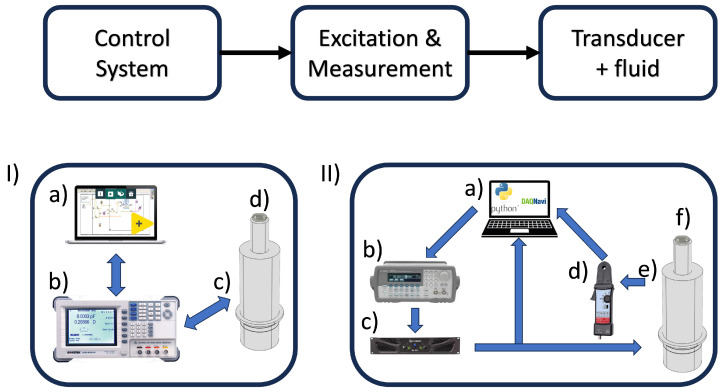
Schematic diagram of the experimental setup. The upper part illustrates a block diagram representing the flow of both experimental systems developed. (**I**) Measurements of fluids at equilibrium state: Experimental system developed to characterize the behavior of glycerol, polyethylene glycol, and BSA protein molecules at different concentrations: (a) computer with software developed in Labview; (b) GW-Instek LCR meter, model 8101G; (c) homemade 30 kHz resonant ultrasonic transducer based on the Langevin piezoelectric sandwich design; (d) fluid to be characterized. (**II**) Protein unfolding kinetics experiment: Experimental system developed to characterize the interaction kinetics of BSA protein with urea: (a) Python software to control the function generator and Advantech model PCIE-1840L high-speed acquisition card; (b) Agilent model 33220A function generator; (c) SKP model MAX300x power amplifier to feed the generated signal; (d) Pintek current probe model PA-699 to record current flow through the transducer; (e) homemade 30 kHz resonant ultrasonic transducer based on the Langevin piezoelectric sandwich design; (f) fluid to be characterized.

The experimental setup designed to characterize the fluids is illustrated in Figure 1. In this setup, a homemade 30 kHz resonant ultrasonic transducer based on the Langevine Langevin piezoelectric sandwich design [24] serves as the main exploratory element to perform the measurements. To accurately determine the behavior of the transducer, it is essential to record both the voltage signal and the current flowing through it. These parameters are crucial for establishing the frequency response of the transducer and identifying any changes resulting from its interaction with the fluid on its exposed surface. To achieve this objective, two experimental designs were conceptualized, each with a configuration represented in the upper part of Figure 1. An integral part of these designs is a frequency and voltage control system, along with the signal application stage, which together facilitate the accurate measurement of both the transducer’s response and its interaction with the fluid.

#### 2.1.1. Measurements of Fluids at the Equilibrium State

To investigate the behavior of different molecular solutions (glycerol, PEG 8000, and BSA protein), the first experimental design was proposed to offer a detailed frequency response, including both the phase and impedance of the transducer, to various concentrations of these fluids (see Figure 1I). A GW-Instek LCR meter, model 8101G, controlled by proprietary software developed in LABVIEW (https://www.ni.com/en/support/downloads/software-products/download.labview.html#521715, accessed on 22 May 2024) and connected via GPIB, was utilized, An W-Instek LCR model 8101G controlled by proprietary software developed in LABVIEW, connected through GPIB, was used.

This software enables the adjustment of the excitation frequency while maintaining a constant voltage of 2 V, allowing for analysis within the frequency range surrounding the resonance of the transducer. The measurement was carried out in a range of 100 Hz with 0.2 Hz steps, using an integration time of 200 ms at each frequency to ensure maximum sensitivity. While this system lacks direct voltage and current measurements, it does offer phase and impedance data for every required frequency. However, it is worth noting that to cover a range of 100 Hz with 0.2 Hz steps, the system requires approximately 6 min.

This procedure is repeated three times for each concentration. This time constraint restricts the ability to monitor the kinetics of molecules effectively. The experiments were performed under room-temperature conditions.

#### 2.1.2. Protein Unfolding Kinetics Experiment

To analyze the kinetics of the interaction between BSA protein and urea (a chaotropic agent), it is necessary to obtain detailed phase data. Additionally, impedance and frequency-dependent quality factor data must also be obtained for each set of measurements. This provides a complete understanding of transducer behavior. The analysis is conducted under different operating conditions. For this purpose, a second experimental design is proposed (see Figure 1II). This design is based on a control program developed in Python 3.7.0. The program allows for variation in both the excitation voltage and the frequency. It utilizes an Agilent function generator, model 33220. The function generator is connected to the controller computer via USB. The generated signal is amplified and then used to excite the transducer under study. With the use of a current probe, it is possible to measure the current flowing through the transducer. Both the excitation voltage signal and the current are acquired using a high-speed acquisition card. The card, an Advantech model PCIE-1840L, is connected to the computer via PCI Express. This experimental system can conduct up to 500 measurements per second, allowing it to sweep 100 Hz in 1 s with a step size of 0.2 Hz. A sample volume of 500 μL is used, and 7 curves are taken over a total duration of 3000 s for each molecule. The experiments were performed under room-temperature conditions.

To analyze the kinetics of the interaction between BSA protein and urea (chaotropic agent), it is necessary to obtain detailed phase, impedance, and frequency-dependent quality factor data for each set of measurements, providing a complete understanding of transducer behavior under different operating conditions. For this purpose, a second experimental design (see Figure 1II) based on a control program developed in Python, which allows one to vary both the excitation voltage and frequency through an Agilent function generator, model 33220, is connected to the controller computer via USB. The generated signal is amplified before exciting the transducer under study. With the use of a current probe, it is possible to measure the current flowing through the transducer. Both the excitation voltage signal and the current are acquired using a high-speed acquisition card, Advantech model PCIE-1840L, connected to the computer via PCI Express. This experimental system can conduct up to 500 measurements per second, allowing it to sweep 100 Hz in 1 s with a step size of 0.2 Hz.

### 2.2. Data Acquisition

#### 2.2.1. Measurements of Fluids in an Equilibrium State

To elucidate the transducer response, the changes in impedance and phase as a function of frequency for each fluid concentration were acquired. The study utilized three molecular solutions, using the experimental setup in Figure 1I): glycerol at 95% (DB Difco), creating diluted solutions ranging from 10% to 40% *v*/*v* by mixing with Milli-Q water.

In a similar way, we prepared solutions of Polyethylene Glycol 8000 (PEG 8000, Bioworld) at concentrations of 10% to 40% *v*/*v*, using Milli-Q water as the diluent, following the methodology outlined by Gonzalez et al. [25].

This choice was informed by the distinctive rheological properties of each compound; glycerol demonstrates relatively minor variations in viscosity with increased concentration, whereas PEG 8000 exhibits marked viscosity changes under similar conditions. The density and viscosity values of glycerol used in this study were obtained from the book of the Glycerine Producers Association [26], utilizing their values at a temperature of T = 25 °C. Meanwhile, the density and viscosity data for PEG 8000 were obtained from [25] at a temperature of T = 25 °C.

**Figure A1:**
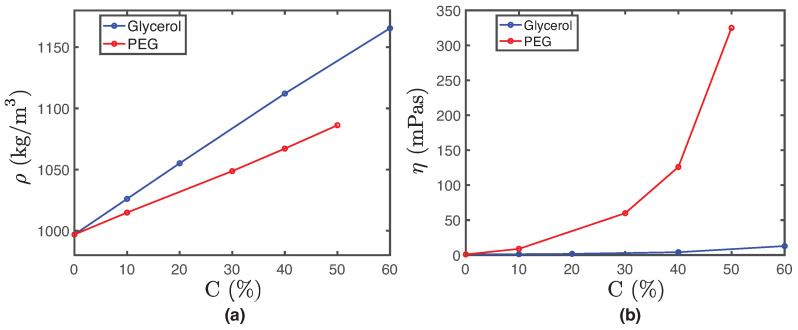
Density ρ and viscosity η as a function of concentration. (**a**) Relation of density ρ as a function of concentration of glycerol and PEG 8000. (**b**) Relation of viscosity η as a function concentration of glycerol and PEG 8000.

Both the density and viscosity of a fluid can vary depending on temperature and pressure [27]. For water, it can be observed that within a range of ±5 °C around the ambient temperature, the viscosity can vary by 20% while the density can vary by 0.21% [28]. In the case of PEG, the viscosity depends on both temperature and concentration [25], as it does in BSA solutions, where similar behavior has been reported [29]. For this reason, it is necessary to consider the temperature when carrying out the experiments, estimating the possible variations in viscosity and density attributable to this process so they are not confused with variations due to other phenomena of interest. This contrast provides a comprehensive framework for evaluating the sensor’s sensitivity and accuracy across a spectrum of viscous environments. See Figure A1.

Finally, bovine serum albumin (BSA, 250 mg/mL, Merck, Santiago, Chile) was prepared at concentrations between 0 and 250 mg/mL, as shown in Figure A3.

A 500 μL droplet was placed for each molecular fluid and for each concentration analyzed. Measurements were taken over a specific frequency range, close to the resonant frequency, with an acquisition time of 6 min. Subsequently, the droplet was replaced with another of the same concentration, and the measurement was repeated. This procedure was repeated three times for each molecular fluid concentration (for more information take a look at Supplementary Materials Figures S1–S3).

#### 2.2.2. Protein Unfolding Kinetics Experiment

To study the unfolding of the BSA protein, a droplet of 500 µL was used to carry out the following experiments, using the experimental setup in Figure 1II.

The experiments included a water control, with a drop of Milli-Q water; urea control, with a drop of the final concentration of 5.7 M urea, diluted in Milli-Q water from the 7 M urea stock solution; BSA Control, with a drop of final concentration of 50 mg/mL BSA, diluted in Milli-Q water from the 250 mg/mL BSA stock solution; and BSA plus Urea kinetics experiment, with a drop of final concentration of 5.7 M urea and 50 mg/mL BSA.

In each experiment, the droplet was deposited on the resonant ultrasonic transducer. Measurements were taken over a specific frequency range near the resonance of the transducer, with an acquisition time of 10 s per scan and 0.2 Hz steps for a total of 300 scans per droplet (for more information take a look at Supplementary Materials Figures S1–S3). Thus, the total acquisition time was 50 min. The data were obtained directly from the setup included impedance and phase, both as a function of the previously mentioned frequency range. In all kinetics experiments, an image of the drop was obtained as a function of time to correlate the transducer parameters with the volume of fluid above it. The droplet was recorded laterally with the Pixelink 741 camera during the experiment described in Section 2.2, capturing an image of 1280 × 1024 pixels at the end of each scan, every 10 s.

### 2.3. Data Analysis

#### 2.3.1. Transducer Parameters

**Figure 2:**
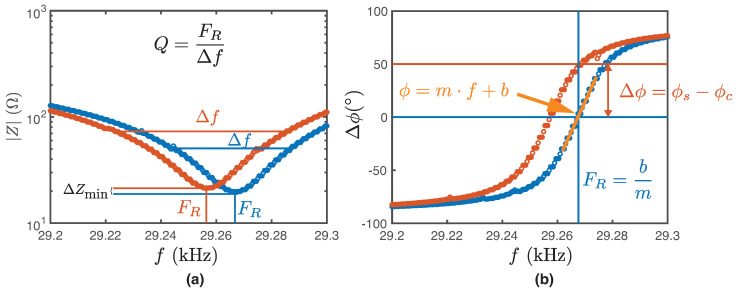
Transducer parameters. Graphical representation of the four transducer parameters that are sensitive to fluid properties. (**a**) In the impedance |Z| versus frequency *f* graph, $\Delta F_{R}$ is obtained, which allows the calculation of the quality factor ΔQ for each curve. The minimum impedance $\Delta Z_{min}$ corresponds to the difference between the minimum of the red curve and the control curve (blue). (**b**) The phase ϕ is obtained by finding the phase value (yellow line) at the resonance frequency $F_{R}$ of the control. The phase difference Δϕ is obtained by finding the phase value at the resonance frequency of the control.

Four transducer response parameters were defined and calculated for the interaction with the different fluids:

- **Resonance frequency ($F_{R}$):** To obtain the resonance frequency, a linear fit is performed on the phase versus frequency curve. In the equation of the obtained line (ϕ=m·f+b), the resonance frequency is when the phase is equal to zero ($F_{R}=-b/m$). The delta of the resonance frequency, $\Delta F_{R}$, is the difference between the resonance frequency and the resonance frequency of the control. See Figure 2a).
- **Phase difference (Δϕ):** The phase difference is obtained by finding the phase value at the resonance frequency of the control. See Figure 2b).
- **Minimum Impedance Difference ($\Delta Z_{min}$):** The minimum value of the impedance curve is obtained, and it is subtracted in each case with respect to the control. See Figure 2a).
- **Quality factor difference (ΔQ):** The ratio between the resonance frequency $F_{R}$ and the full width at half maximum (FWHM) (Δf). See Figure 2.

To analyze the variation in these parameters for each concentration of the fluid, the Δ variations were considered, comparing each case with the same parameter obtained when the fluid is only Milli-Q water.

As indicated above, each experiment was carried out in triplicate, so an average ($\bar{x}$), and the error to student’s t-distribution with a 90% confidence interval. The number of each data point, in our case, is *n* = 3. The error bar corresponds to this value.

#### 2.3.2. Analysis of the Drop Image to Obtain the Volume as a Function of Time

**Figure 3:**
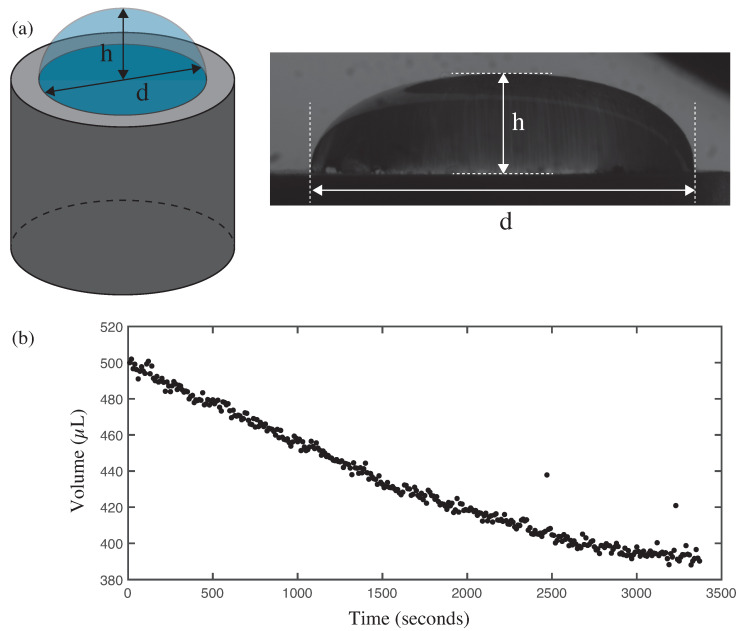
Milli-Q water drop analysis. (**a**) (left) Scheme of the experimental setup: h corresponds to the maximum height of the water drop with respect to the surface of the ultrasonic transducer, and d to the instantaneous diameter of the drop. Both quantities vary throughout the experiment. (right) Photo of the experimental setup. (**b**) Evolution over time of water drop volume during the experiment.

The dynamics of the experiment are captured through photographs taken of the Milli-Q water droplet profile every 10 s. Through image analysis of each photograph, the maximum height (*h*) (with respect to the substrate) and the instantaneous diameter (*d*) of the drop are measured (see Figure 3a). With this information, the variation in the volume of the water drop can be calculated using the following formula (1), which corresponds to the volume of a sector of a sphere [30]:

(1) $$V=\frac{\pi }{6}\left[3{\left(\frac{d}{2}\right)}^{2}+h^{2}\right]h$$

The parameters *h* and *d* decrease with time, so it is reasonable to observe that the volume of the drop also decreases (see Figure 3b).

To reduce the noise in the curves obtained for different molecules as a function of time (water, urea, BSA, and BSA plus urea), a moving average filter is applied every 30 data points (5 min).

## 3. Results and Discussion

To comprehend the alterations in the transducer’s response caused by changes in the physical parameters of the solvent, we examine the $\Delta F_{R}$, Δϕ, $\Delta Z_{min}$, and ΔQ values corresponding to different density (ρ) and viscosity (η) levels.

**Figure 4:**
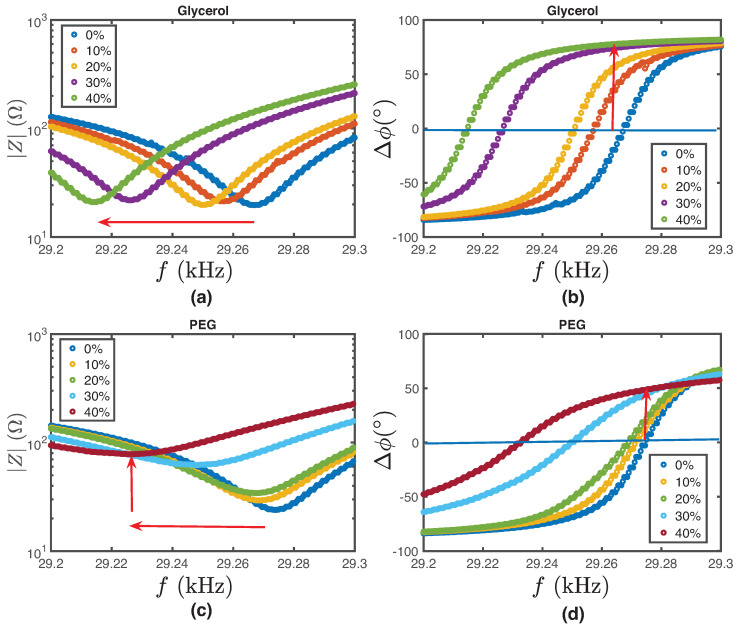
Experimental curves for glycerol and PEG. The concentrations of the diluted glycerol solutions are represented as follows: 0% (Milli-Q water) by blue points, 10% by red points, 20% by yellow points, 30% by purple points, and 40% by green points. (**a**) Impedance versus frequency curves for glycerol. The red arrow indicates the leftward shift of the curves as the concentration increases. (**b**) Phase versus frequency curves for 95% glycerol. The concentrations of the diluted PEG solutions are represented as follows: 0% (Milli-Q water) by blue points, 5% by red points, 10% by yellow points, 20% by purple points, 30% by green points, and 40% by light blue points. The red arrow indicates the upward and leftward shift as the concentration increases. (**c**) Impedance versus frequency curves for PEG. The red arrow indicates the leftward shift of the curves as the concentration increases. (**d**) Phase versus frequency curves for PEG. The red arrow indicates the upward and leftward shift as the concentration increases.

The results obtained from the experimental study described in the previous section are presented herein. Figure 4 illustrates the characterization of glycerol and PEG solution samples, where we explore the impedance and phase-versus-frequency curves as functions of the substances at different concentrations. For glycerol, concentrations were varied and are represented by different colors: 0% (pure Milli-Q water) is shown in blue, along with increasing concentrations of 10%, 20%, 30%, and 40%. It was observed that as the glycerol concentration increased, there was a noticeable leftward shift in the impedance versus frequency curves, indicated by a red arrow. The phase versus frequency curves also demonstrate a shift, although the direction is not specified. Similarly, PEG solutions were examined and are coded as follows: 0% (pure Milli-Q water) is shown in blue, along with increasing concentrations of 10%, 20%, 30% and 40%. For PEG, both the impedance and phase versus frequency curves exhibit a leftward shift as the PEG concentration increases, with the phase curves also showing an upward trend, again highlighted by red arrows. This comprehensive analysis provides valuable insights into how varying concentrations of glycerol and PEG affect their electrical behavior, with shifts in impedance and phase frequency curves serving as indicators of these changes, which can be correlated to variations in the density and viscosity of the sample solutions. The variation in parameters such as resonance frequency, by adding mass to its surface, has already been explored in previous works [31], where the effect of virtual added mass-induced changes in the resonant frequency of a vibrating structure has been appreciated. This effect is attributed to the fluid density variation experienced when changing the concentration, in this case, glycerol or PEG.

Regarding the observed variations in minimum system impedance, it is worth noting that this effect has been previously reported in other resonant systems. For example, Saluja et al. [32] showed that this parameter varies depending on the product of density and solvent viscosity when using quartz crystal impedance analysis in sample volumes of microliters. Jia et al. [33] were able to measure viscosity variations by detecting changes in impedance caused by resonant excitation of a system at high volumes (around 10 mL). The results indicate a significant variation in resonance frequency with changes in fluid concentration, specifically with increasing concentrations of glycerol and PEG. Additionally, an increase in PEG concentration results in variations in minimum impedance.

Due to this observation, we will decouple the density and viscosity variables to understand their individual contributions to the different electromechanical parameters.

### 3.1. Representative Transducer Parameters for Density

**Figure 5:**
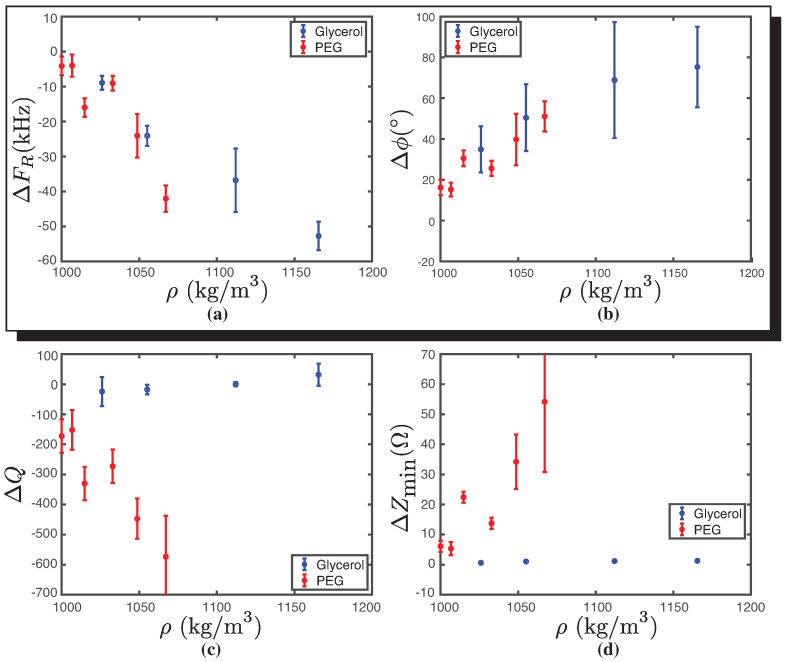
Behavior of the four transducer parameters as a function of density for glycerol (blue) and PEG (red). (**a**) Resonance frequency ($\Delta F_{R}$) as a function of density variation (ρ). For both molecules, a decrease in $\Delta F_{R}$ is observed as ρ increases. (**b**) Phase difference (Δϕ) as a function of density variation (ρ). For both molecules, an increase in Δϕ is observed as ρ increases. The parameters $\Delta F_{R}$ and Δϕ clearly show variations at different densities. (**c**) Quality factor difference (ΔQ) as a function of density (ρ) variation. For glycerol, the behavior of ΔQ remains constant, unlike PEG, which decreases as ρ increases. (**d**) Minimum impedance difference ($\Delta Z_{min}$) as a function of density variation (ρ). In this case, the parameter exhibits the same behavior described earlier. The error bar corresponds to Student’s t-distribution with a 90% confidence interval. The number of each data point, in our case, is *n* = 3.

Figure 5 summarizes the critical parameters obtained from Figure 4 as a function of solution sample density. The density and viscosity values were obtained from [25,26] as a function of the concentration of PEG and glycerol, respectively.

Figure 5a shows that as the density of fluids increases, the difference in resonance frequency compared to water decreases, exhibiting a similar behavior for both fluids. The range of densities obtained for both fluids is comparable due to the concentrations used.

In Figure 5b, we can observe that Δϕ increases as the solution’s density increases. The increase in Δϕ with density suggests changes in the wave propagation speed or alterations in the medium’s viscoelastic properties, offering a valuable parameter for studying material behavior under varying density conditions.

Figure 5c,d illustrate the difference in the quality factor (ΔQ) and the minimum impedance ($\Delta Z_{min}$) as a function of liquid density, revealing different behaviors between glycerol and PEG, unlike the previously studied parameters. In both cases, the behavior of glycerol as a function of density is approximately constant, while for PEG, a decreasing function is observed for the quality factor and an increasing function for the minimum impedance. It is observed that the parameters $\Delta F_{R}$ and Δϕ are reliable indicators to describe the density behavior for the two molecules studied. This is due to the fact that, regardless of the type of fluid, similar behaviors are obtained for similar densities.

### 3.2. Representative Transducer Viscosity Parameters

**Figure 6:**
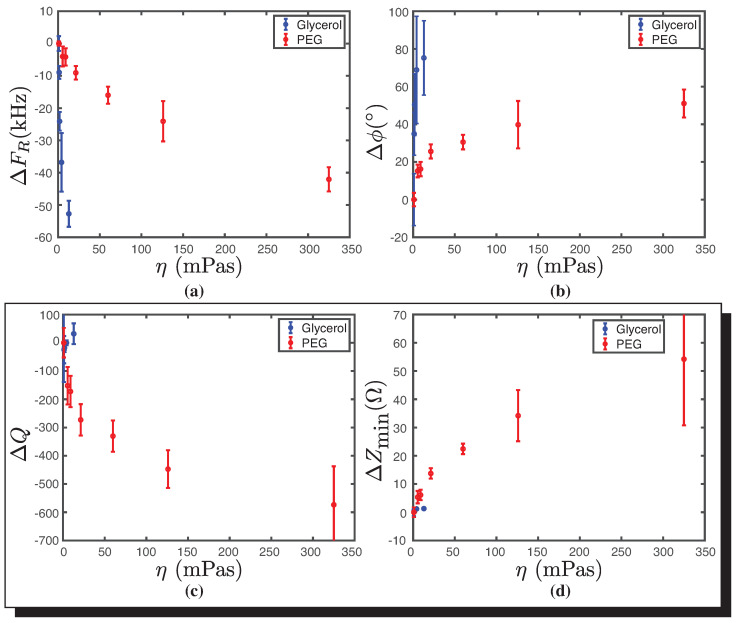
Behavior of the four transducer parameters as a function of viscosity (η) for glycerol (blue) and PEG (red). (**a**) Resonance frequency ($\Delta F_{R}$) as a function of viscosity (η). (**b**) Phase difference (Δϕ) as a function of viscosity (η). (**c**) Quality factor difference (ΔQ) as a function of viscosity (η). In the inset plot with η on a logarithmic scale, it is observed that for glycerol and PEG, both curves present similar behaviors. (**d**) Minimum impedance difference ($\Delta Z_{min}$) as a function of viscosity (η). In the inset plot with η on a logarithmic scale, it is observed that for glycerol and PEG, both curves present similar behaviors. The error bar corresponds to Student’s t-distribution with a 90% confidence interval. The number of each data points, in our case, is *n* = 3.

Figure 6 depicts the critical parameters as a function of viscosity. It is evident that PEG experiences a significant increase in viscosity as the concentration rises. This suggests that the critical parameter remains nearly unchanged for small viscosity changes. Furthermore, it is noticeable that the behavior of $\Delta F_{R}$ decreases linearly and dramatically for glycerol, while showing a less steep decline for PEG (see Figure 6a). The Δϕ exhibits a nonlinear dependence on viscosity, with a pronounced increase for glycerol. Conversely, the difference in quality factor and the change in minimal impedance are nearly insensitive to changes in viscosity for glycerol, as shown in Figure 6c and Figure 6b, respectively, with a stronger dependence observed for changes in PEG viscosity. Overall, the increase in viscosity at the molecular level alters the acoustic properties of a material, influencing the attenuation, propagation speed, impedance, and frequency response of ultrasonic waves as they interact with the medium [34,35].

Similarly, the behavior of the four parameters as a function of the viscosity of glycerol and PEG is plotted.

For the parameters $\Delta F_{R}$ and Δϕ, the behaviors for both molecules are different in similar viscosity ranges under 50 mPas and are therefore not representative of viscosity. For ΔQ, a variation is observed for large values of viscosity, such as those obtained for the PEG concentrations used. A similar behavior is observed for $\Delta Z_{min}$, which varies significantly between 50 mPas and 350 mPas. This same trend is observed in Figure A1b for the relationship between viscosity and glycerol-PEG concentration. Because of these results, the parameters ΔQ and $\Delta Z_{min}$ are good candidates to represent the viscosity of the medium. In the literature [32,36,37,38], it is observed that the parameters of density and viscosity are analyzed in a coupled manner (η·ρ), as they correspond to a classic electromechanical analogy. In this case, we analyze them separately to understand the contribution of each.

### 3.3. Characterization of the BSA Denaturation

**Figure 7:**
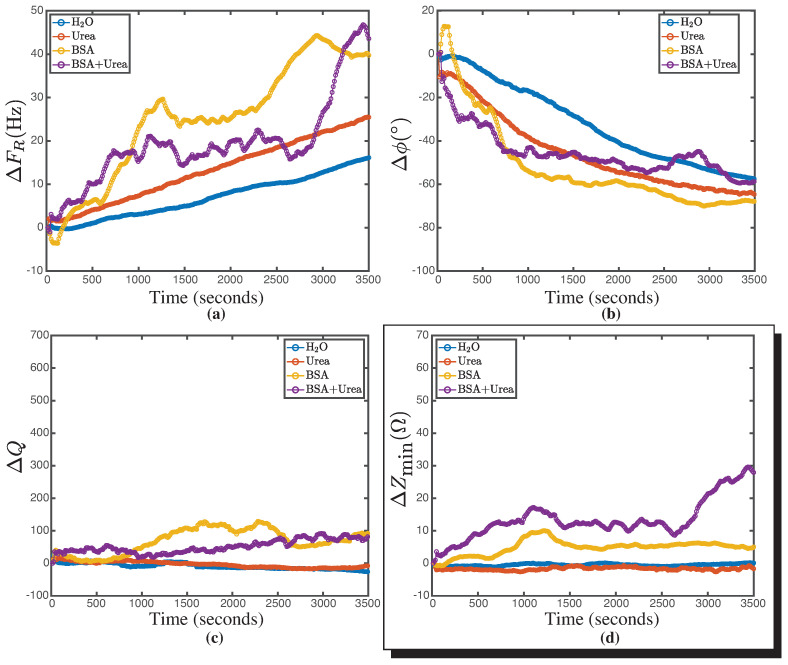
Unfolding kinetics of the four transducer parameters as a function of time (t) for Milli-Q water (H_2_O) (blue), 5.7 M urea (red), 50 mg/mL BSA (yellow), and 5.7 M urea + 50 mg/mL BSA (purple). (**a**) Resonance frequency ($\Delta F_{R}$) as a function of time (t). (**b**) Phase difference (Δϕ) as a function of time (t). (**c**) Quality factor difference (ΔQ) as a function of time (t). (**d**) Minimum impedance difference ($\Delta Z_{min}$) as a function of time (t). For both urea and water, the behavior is constant over time. In the case of BSA over time, it remains constant with a variation at 1000 s. When observing BSA with urea, a difference is noticed from the previous curve. After 3000 s, the unfolding effect of BSA in the bulk due to urea begins to be apparent. Each experiment was carried out in triplicate.

**Figure A2:**
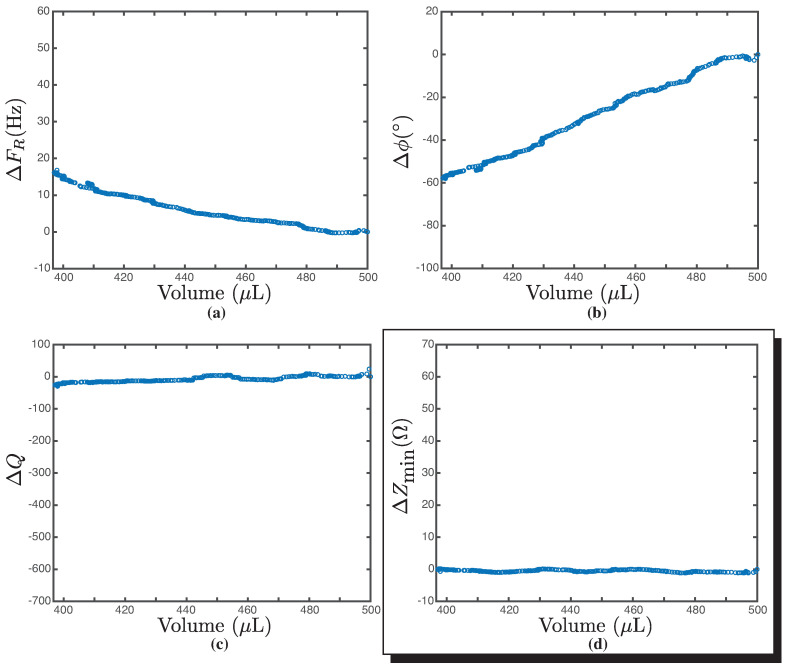
Behavior of the four transducer parameters as a function of volume (*V*) for Milli-Q water. (**a**) Resonance frequency ($\Delta F_{R}$) as a function of volume (*V*). (**b**) Phase difference Δϕ as a function of volume (*V*). (**c**) Quality factor difference as a function of volume (*V*). (**d**) Minimum impedance difference $\Delta Z_{min}$ as a function of volume (*V*).

**Figure A3:**
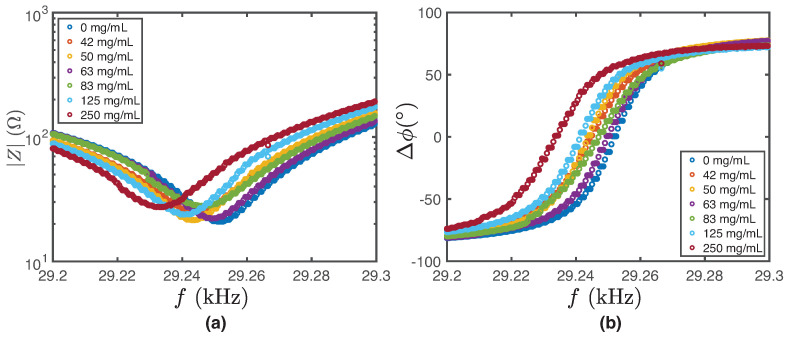
BSA concentrations are represented as follows: 0 mg/mL (Milli-Q water) by blue points, 42 mg/mL by red points, 50 mg/mL by yellow points, 63 mg/mL by purple points, 83 mg/mL by green points 125 mg/mL by light blue points and 250 mg/mL by burgundy points. (**a**) Impedance versus frequency curves are observed for BSA. (**b**) Phase versus frequency curves are observed for BSA.

To explore a biological application involving viscosity variations without significant changes in density, the denaturation of BSA molecules was chosen. This is because the unfolding of proteins causes considerable variations in the viscosity of solutions [39]. To investigate the sensitivity of the technique, the kinetics of BSA unfolding under a constant concentration of urea was explored. Figure 7a shows the relationship between resonance frequency and time for different molecules, providing a foundation for understanding the device’s response to biological substances. The resonance frequency kinetics data for water and urea show a linear increase in resonance frequency over time. This is expected due to the volume variations that the sample may experience in the current experimental setup, as seen in Figure A2a. The resonance frequency from Figure 5a shows a variation in trend when the PEG concentrations are higher, indicating that when the viscosity of the medium increases, both density and viscosity may not be uncoupled in this parameter. The resonance frequency from Figure 5a shows a variation in the trend when the PEG concentrations are higher, that is, when the viscosity of the medium increases, so it is possible that both variables, density and viscosity, are not uncoupled in this parameter. Figure 7b shows the relationship between Δϕ and time. In this case, the behavior of the curves is similar and consistent with previously reported volume variations as a function of time.

Figure 7c shows the relationship between ΔQ and time. For water and urea, both behaviors are similar and consistent with the previously reported volume variations. However, for BSA and BSA plus urea, fluctuations are observed that may correlate with variations in resonance frequency over the same time interval. Finally, Figure 7d shows the relationship between $\Delta Z_{min}$ and time. For water and urea, both behaviors are similar and consistent with the volume variations observed in the fluid (see Appendix A Figure A2). However, for BSA, small fluctuations of the order of 10 ω are obtained, while for BSA plus urea, variations of the order of 30 ω are experienced. In experiments with equilibrium fluids where the viscosity variation is known (see Figure 6), the $\Delta Z_{min}$ parameter experiences variations on the order of 55 ± 15 Ω when the viscosity varied 330 mPas. The variations of $\Delta Z_{min}$ in BSA + urea can be attributed to variations in viscosity. Viscosity variation upon protein unfolding is a problem that has already been addressed in several studies [21,39,40].

**Figure 8:**
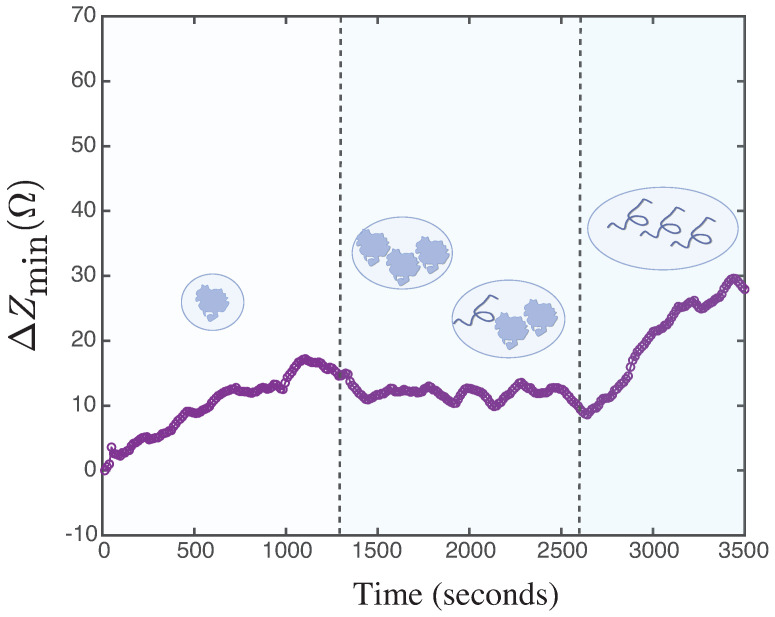
BSA unfolding model in urea. The increase in $\Delta Z_{min}$ observed between times t = 0 and t = 1300 s is linked to the onset of the aggregation process experienced by the BSA molecule. This aggregation leads to a growth effect of the colloid in solution, resulting in a change in the effective viscosity according to the Batchelor–Einstein model for colloidal solutions. Subsequently, a stabilization is observed in $\Delta Z_{min}$ between t = 1300 s and t = 2600 s. Stabilization continues until 2600 s, at which time the system detects changes associated with the unfolding of the BSA molecule interacting with the urea molecule. This interaction generates alterations in the minimum impedance of the system, which are related to changes in the effective viscosity due to the change in the hydrodynamic radius of the colloid.

The viscosity variation can be explained by the change in the volume fraction occupied by the proteins, considered as colloids, in the volume of the solvent [41]. Upon unfolding, the radius of the protein increases, changing this fraction and thus the relative viscosity, which, according to the Batchelor–Einstein relationship [42], depends polynomially on this parameter. The increase in $\Delta Z_{min}$ observed in Figure 7d can be linked to the aggregation process undergone by the BSA molecule, as reported in previous work by Pindrus et al. [43]. This aggregation leads to a growth effect of the colloid in solution, resulting in a change in the effective viscosity according to the Batchelor–Einstein model for colloidal solutions. Subsequently, a stabilization is observed at $\Delta Z_{min}$, which persists until the end of the acquisition time for the BSA solution. In the case of the solution with BSA + urea, this stabilization continues until 2600 s, at which point the system detects changes associated with the unfolding of the BSA molecule interacting with the urea molecule. At that time, urea, as an effective chaotropic agent, forms a direct hydrogen-bonding interaction with BSA through its N-H functional group, resulting in a denaturation process reported by Kumaran [22] and other authors for different proteins [44,45]. This unfolding generates alterations in the minimum impedance of the system, which are related to changes in the effective viscosity due to variations in the hydrodynamic radius of the colloid, as shown in Figure 8.

Although the graph in Figure 7d provides sufficient information to propose the model presented in Figure 8, the other parameters analyzed in the kinetics reveal significant interactions that are worth exploring, such as the attenuation of the BSA signal in the presence of urea in the $\Delta F_{R}$ graph around 1300 s and then at 2800 s.

## 4. Conclusions

The initial approach to characterizing solutions has been successfully achieved using a transducer drop system, which uses a smaller sample volume (500 μL) non-destructively and improves acquisition time, achieving a data collection rate of 500 data points per second. The electromechanical response of the transducer, encapsulated by four parameters, resonance frequency, phase shift, quality factor, and minimum impedance, has been correlated with variations in glycerol and PEG concentration. We observed consistent patterns for density in the resonance frequency and phase difference parameters and for viscosity in the minimum impedance and quality factor parameters.

Serving as a proof of concept and projecting its use as a sensor for detecting activity or conformational changes in biologically relevant biomolecules, we noted a change in the minimum impedance of the transducer drop system over time when BSA was exposed to a high concentration of urea. In this scenario, the protein is known to unfold. These findings align with the anticipated changes in the hydrodynamic radius of the protein, thereby affecting the viscosity of the solution. Future enhancements to the system are underway, along with the development of resonant transducers capable of probing even smaller sample volumes. Work is being conducted on alternative models to the classical electromechanical ones to independently isolate the contributions of each parameter. These improvements aim to refine the methodology for quantifying protein activity, coagulation phenomena, or any biological system where timely viscosity quantification is crucial.

## Data Availability

The original contributions presented in the study are included in the article, further inquiries can be directed to the corresponding authors.

## Supplementary Materials

The following are available online at https://www.mdpi.com/article/10.3390/bios14070346/s1, Figure S1: Impedance vs. glycerol concentrations; Figure S2: Impedance vs. PEG concentrations; Figure S3: Impedance vs. different fluids.

## Abbreviations

The following abbreviations are used in this manuscript:

η	viscosity
ρ	density
PEG	Polyethylene Glycol 8000
BSA	Bovine serum albumin
FWHM	full width at half maximum
$F_{R}$	Resonance frequency
Δϕ	Phase difference
$\Delta Z_{min}$	Minimum Impedance Difference
ΔQ	Quality factor difference
*s*	seconds

## References

[1] Badidi Bouda A., Benchala K., Alem K. (2000). Ultrasonic characterization of materials hardness. Ultrasonics.

[2] Leclaire P., Kelders L., Lauriks W., Melon M., Brown N., Castagnede B. (1996). Determination of the viscous and thermal characteristic lengths of plastic foams by ultrasonic measurements in helium and air. J. Appl. Phys..

[3] Doyle P., Scala C.M. (1978). Crack depth measurement by ultrasonics: A review. Ultrasonics.

[4] Salinas V., Vargas Y., Ruzzante J., Gaete L. (2010). Localization algorithm for acoustic emission. Phys. Procedia.

[5] Carovac A., Smajlovic F., Junuzovic D. (2011). Application of Ultrasound in Medicine. Acta Inf. Med..

[6] Al-Dhabyani W., Gomaa M., Khaled H., Fahmy A. (2020). Dataset of breast ultrasound images. Data Brief.

[7] Torres H.R., Morais P., Oliveira B., Birdir C., Rüdiger M., Fonseca J.C., Vilaça J.L. (2022). A review of image processing methods for fetal head and brain analysis in ultrasound images. Comput. Methods Programs Biomed..

[8] Yao Y., Pan Y., Liu S. (2020). Power ultrasound and its applications: A state-of-the-art review. Ultrason. Sonochem..

[9] Abdulkareem A., Erturun U., Mossi K. (2020). Non-Destructive Evaluation Device for Monitoring Fluid Viscosity. Sensors.

[10] Kazys R., Sliteris R., Rekuviene R., Zukauskas E., Mazeika L. (2015). Ultrasonic Technique for Density Measurement of Liquids in Extreme Conditions. Sensors.

[11] Hong T., Iwashita K., Shiraki K. (2018). Viscosity control of protein solution by small solutes: A review. Curr. Protein Pept. Sci..

[12] Puneeth S.B., Kulkarni M.B., Goel S. (2021). Microfluidic viscometers for biochemical and biomedical applications: A review. Eng. Res. Express.

[13] Ma X., Zhang X., Zhang B., Yang D., Sun H., Tang Y., Shi L. (2024). Dual-responsive fluorescence probe for measuring ${\mathrm{HSO}}_{3}^{-}$
and viscosity and its application in living cells and real foods. Food Chem..

[14] Zidar M., Rozman P., Belko-Parkel K., Ravnik M. (2020). Control of viscosity in biopharmaceutical protein formulations. J. Colloid Interface Sci..

[15] Zhang Z., Liu Y. (2017). Recent progresses of understanding the viscosity of concentrated protein solutions. Curr. Opin. Chem. Eng..

[16] Moino C., Artusio F., Pisano R. (2023). Shear stress as a driver of degradation for protein-based therapeutics: More accomplice than culprit. Int. J. Pharm..

[17] Ashton L., Dusting J., Imomoh E., Balabani S., Blanch E.W. (2010). Susceptibility of different proteins to flow-induced conformational changes monitored with Raman spectroscopy. Biophys. J..

[18] Bekard I.B., Asimakis P., Teoh C.L., Ryan T., Howlett G.J., Bertolini J., Dunstan D.E. (2012). Bovine serum albumin unfolds in Couette flow. Soft Matter.

[19] Jaspe J., Hagen S.J. (2006). Do protein molecules unfold in a simple shear flow?. Biophys. J..

[20] Hobson E.C., Li W., Juliar B.A., Putnam A.J., Stegemann J.P., Deng C.X. (2021). Resonant acoustic rheometry for non-contact characterization of viscoelastic biomaterials. Biomaterials.

[21] Munoz R., Aguilar-Sandoval F., Bellon L., Melo F. (2017). Detecting protein folding by thermal fluctuations of microcantilevers. PLoS ONE.

[22] Kumaran R., Ramamurthy P. (2011). Denaturation mechanism of BSA by urea derivatives: Evidence for hydrogen-bonding mode from fluorescence tools. J. Fluoresc..

[23] Bramanti E., Ferrari C., Angeli V., Onor M., Synovec R.E. (2011). Characterization of BSA unfolding and aggregation using a single-capillary viscometer and dynamic surface tension detector. Talanta.

[24] Amir A., Pak S., Abbas M.A. (2009). An approach to design a high power piezoelectric ultrasonic transducer. J. Electroceramic.

[25] Gonzalez-Tello P., Camacho F., Blazquez G. (1994). Density and Viscosity of Concentrated Aqueous Solutions of Polyethylene Glycol. J. Chem. Eng. Data.

[26] Glycerine Producers Association (1963). Physical Properties of Glycerine and Its Solutions. Glycerine Producers’ Association. https://books.google.cl/books?id=XpeaGQAACAAJ.

[27] Likhachev E.R. (2003). Dependence of water viscosity on temperature and pressure. Tech. Phys..

[28] Wagner W., Kretzschmar H.J. (2014). International Steam Tables.

[29] Masuelli M.A. (2013). Study of Bovine Serum Albumin Solubility in Aqueous Solutions by Intrinsic Viscosity Measurements. Adv. Phys. Chem..

[30] Fan C.F., Cagin T. (1995). Wetting of crystalline polymer surfaces: A molecular dynamics simulation. J. Chem. Phys..

[31] Roy K., Gupta H., Shastri V., Dangi A., Jeyaseelan A., Dutta S., Pratap R. (2019). Fluid density sensing using piezoelectric micromachined ultrasound transducers. IEEE Sens. J..

[32] Saluja A., Kalonia D.S. (2004). Measurement of fluid viscosity at microliter volumes using quartz impedance analysis. Aaps Pharmscitech.

[33] Jia S., Luo M. (2021). Monitoring of liquid viscosity for viscous dampers through a wireless impedance measurement system. Appl. Sci..

[34] Garret S.L. (2020). Understanding Acoustic: An Experimentalist’s View of Sounds and Vibration.

[35] Prugne C.H., Van Est J., Cros B., Léveque G., Attal J. (1998). Measurement of the viscosity of liquids by near-field acoustics. Meas. Sci. Technol..

[36] Wang F., Zhang H., Liang C., Tian Y., Zhao X., Zhang D. (2016). Design of High Frequency Ultrasonic Transducers with Flexure Decoupling Flanges for Thermosonic Bonding. IEEE Trans. Ind. Electron..

[37] Zhang J.-G., Long Z.-L., Ma W.-J., Hu G.-H., Li Y.-M. (2019). Electromechanical Dynamics Model of Ultrasonic Transducer in Ultrasonic Machining Based on Equivalent Circuit Approach. Sensors.

[38] Bybi A., Atlas N.E., Drissi H., Garoum M., Hladky-Hennion A.-C. (2017). One-dimensional electromechanical equivalent circuit for piezoelectric array elements. Proceedings of the 2017 International Conference on Electrical and Information Technologies (ICEIT).

[39] Kimura S., Komiyama T., Masuzawa T., Yokoya M., Oyoshi T., Yamanaka M. (2023). Bovine serum albumin hydrogel formation: Ph dependence and rheological analyses. Chem. Pharm. Bull..

[40] Tu R.S., Breedveld V. (2005). Microrheological detection of protein unfolding. Phys. Rev. E.

[41] Wagner N.J., Mewis J. (2021). Theory and Applications of Colloidal Suspension Rheology.

[42] Pal R. (2020). New generalized viscosity model for non-colloidal suspensions and emulsions. Fluids.

[43] Pindrus M.A., Cole J.L., Kaur J., Shire S.J., Yadav S., Devendra S. (2017). Kalonia Effect of Aggregation on the Hydrodynamic Properties of Bovine Serum Albumin. Pharm. Res..

[44] Casanova-Morales N., Alavi Z., Wilson C.A.M., Zocchi G. (2018). Identifying Chaotropic and Kosmotropic Agents by Nanorheology. J. Phys. Chem. B.

[45] Gooran N., Kopra K. (2024). Fluorescence-Based Protein Stability Monitoring—A Review. Int. J. Mol. Sci..

