# Supplementary material for: Ultrasonic Sensor: A Fast and Non-Destructive System to Measure the Viscosity and Density of Molecular Fluids
Source: Biosensors (Basel). 2024 Jul 16;14(7):346. doi: 10.3390/bios14070346 (PMC11274559; doi:10.3390/bios14070346)
Supplement: Supplementary file 1 [file biosensors-14-00346-s001.zip › biosensors-3047095-supplementary.pdf]

biosensors

Supplementary Materials

Ultrasonic Sensor: A Fast and Non-Destructive System to Measure the Viscosity and Density of Molecular Fluids

Romina Munoz [1], Juan-Francisco Fuentealba [2], Sebastian Michea [3], Paula A. Santana [4], Juan Ignacio Martinez [5], Nathalie Casanova-Morales [6,*] and Vicente Salinas-Barrera [3,*]

[1] Departamento de Fisica y Quimica, Facultad de Ingenieria, Universidad Autonoma de Chile, Av. Pedro de Valdivia 425, Providencia, Santiago 8900000, Chile; romina.munoz@uautonoma.cl
[2] Escuela de Ingenieria, Universidad Central de Chile, Avda. Santa Isabel 1186, Santiago 8330601, Chile; juan.fuentealba@ucentral.cl
[3] Grupo de Investigacion Aplicada en Robotica e Industria 4.0, Instituto de Ciencias Aplicadas, Facultad de Ingenieria, Universidad Autonoma de Chile, Santiago 7500912, Chile; sebastian.michea@uautonoma.cl
[4] Instituto de Ciencias Aplicadas, Facultad de Ingenieria, Universidad Autonoma de Chile, El Llano Subercaseaux 2801, San Miguel, Santiago 8910060, Chile; paula.santana@uautonoma.cl
[5] Ingenieria Civil Informatica, Facultad de Ingenieria, Universidad Autonoma de Chile, Av. Pedro de Valdivia 425, Providencia, Santiago 8900000, Chile; juan.martinez2@cloud.uautonoma.cl
[6] Facultad de Artes Liberales, Universidad Adolfo Ibanez, Santiago 7941169, Chile
* Correspondence: casa886@gmail.com (N.C.-M.); vicente.salinas@uautonoma.cl (V.S.-B.)

1. Repetitions in measurements with glycerol

All measurements were performed in triplicate. The following graph shows the independent curves related to each of the glycerol concentrations, where the impedance versus frequency curves are indicated in each of the cases.

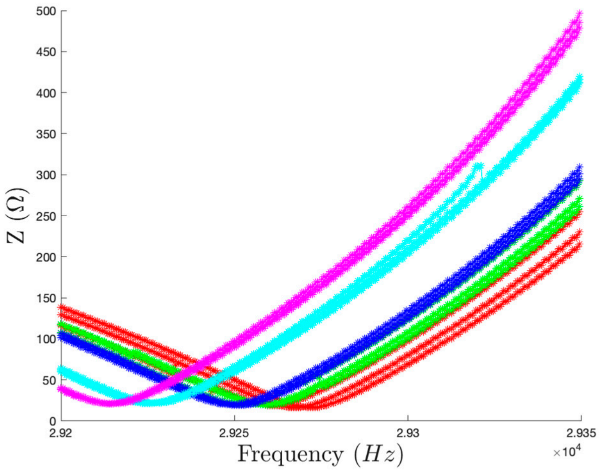

Figure S1. The colors represent the glycerol concentrations in each case. Red 0%, green 10%, blue 20%, cyan 30%, and magenta 40%.

2. Repetitions in measurements with PEG

All measurements were performed in triplicate. The following graph shows the independent curves related to each of the PEG concentrations, where the impedance versus frequency curves are indicated in each of the cases.

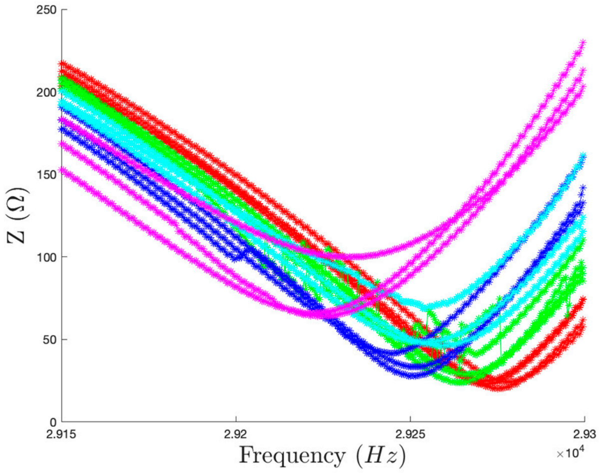

Figure S2. The colors represent the PEG concentrations in each case. Red 0%, green 10%, blue 20%, cyan 30%, and magenta 40%.

3. Characterization of the BSA denaturation.

The following panel of figures displays the acquired signals during the characterization process of different fluids, namely H2O, urea, BSA, and BSA+urea. Each figure contains 300 data sets, with each set comprising 500 data points. These data points represent the variations in the excitation frequency applied to the transducer, which resonates at approximately 30 kHz, and the corresponding response to this excitation. The temporal separation between data sets is 10 seconds.

From these raw data, an analysis was performed to obtain the temporal evolution of four parameters presented in the article: ΔFR (frequency shift), $\Delta\phi$ (phase shift), ΔQ (quality factor change), and $\Delta Zmin$ (minimum impedance change). The methodology for calculating these parameters is detailed in Section 2.3.1 of the article.

Specifically, Figure 7d) of the article was derived by calculating the minimum impedance of each data set and comparing it to the minimum impedance of the initial data set, which corresponds to time zero ($\Delta Zmin$).

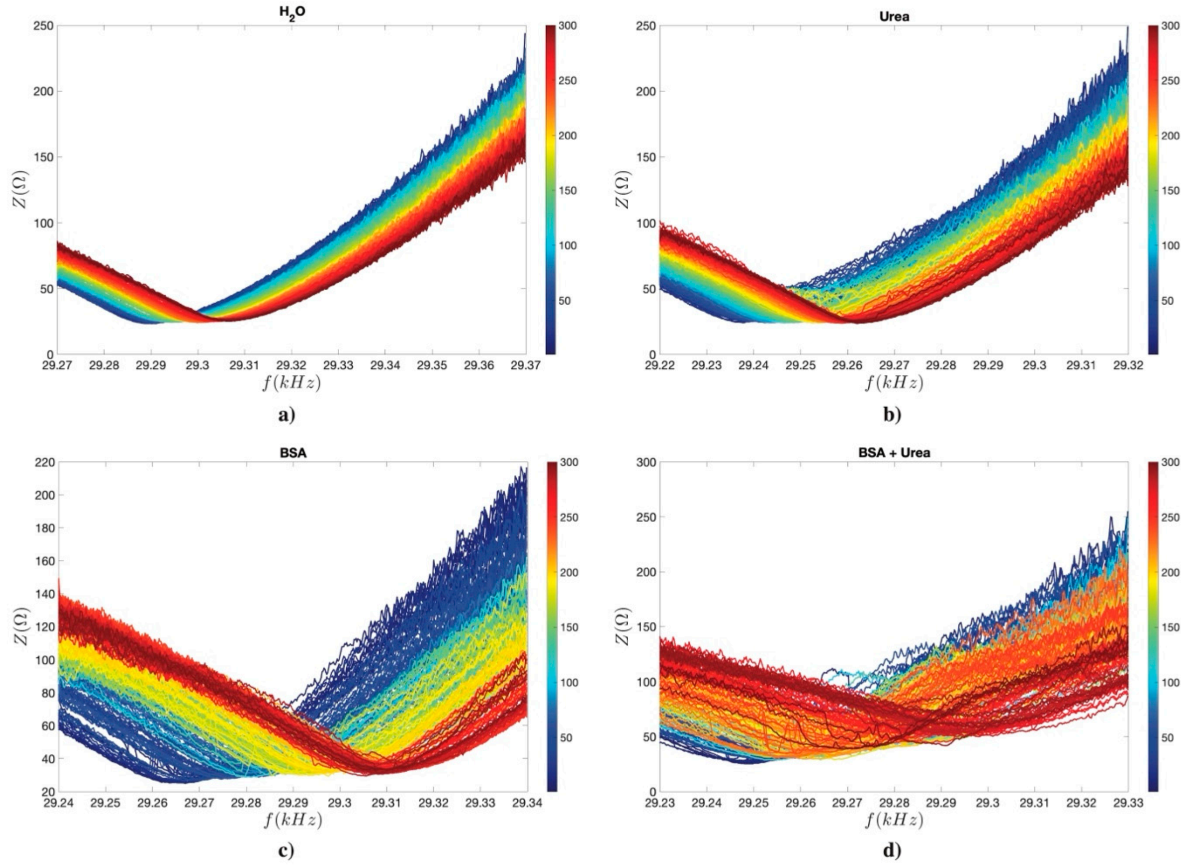

Figure S3. Acquired signals during the characterization process of different fluids: a) H2O; b) urea; c) BSA; d) BSA+urea. Each figure contains 300 data sets, with each set comprising 500 data points.

The data necessary to reproduce the figures in this supplementary material, as well as the figures from the article, can be found in the following repository.

https://github.com/VicenteSalinas/SUV_Biosensors
